# Oscillatory EEG Activity During REM Sleep in Elderly People Predicts Subsequent Dream Recall After Awakenings

**DOI:** 10.3389/fneur.2019.00985

**Published:** 2019-09-20

**Authors:** Serena Scarpelli, Aurora D'Atri, Chiara Bartolacci, Anastasia Mangiaruga, Maurizio Gorgoni, Luigi De Gennaro

**Affiliations:** Department of Psychology, University of Rome “Sapienza”, Rome, Italy

**Keywords:** dream recall, dreaming, elderly, older adults, theta oscillations, REM sleep, EEG, oscillatory activity

## Abstract

Several findings underlined that the electrophysiological (EEG) background of the last segment of sleep before awakenings may predict the presence/absence of dream recall (DR) in young subjects. However, little is known about the EEG correlates of DR in elderly people. Only an investigation found differences between recall and non-recall conditions during NREM sleep EEG in older adults, while—surprisingly—no EEG predictor of DR was found for what concerns REM sleep. Considering REM sleep as a privileged *scenario* to produce mental sleep activity related to cognitive processes, our study aimed to investigate whether specific EEG topography and frequency changes during REM sleep in elderly people may predict a subsequent recall of mental sleep activity. Twenty-one healthy older volunteers (mean age 69.2 ± 6.07 SD) and 20 young adults (mean age 23.4 ± 2.76 SD) were recorded for one night from 19 scalp derivations. Dreams were collected upon morning awakenings from REM sleep. EEG signals of the last 5 min were analyzed by the Better OSCillation algorithm to detect the peaks of oscillatory activity in both groups. Statistical comparisons revealed that older as well as young individuals recall their dream experience when the last segment of REM sleep is characterized by frontal theta oscillations. No Recall (Recall vs. Non-Recall) × Age (Young vs. Older) interaction was found. This result replicated the previous evidence in healthy young subjects, as shown in within- and between-subjects design. The findings are completely original for older individuals, demonstrating that theta oscillations are crucial for the retrieval of dreaming also in this population. Furthermore, our results did not confirm a greater presence of the theta activity in healthy aging. Conversely, we found a greater amount of rhythmic theta and alpha activity in young than older participants. It is worth noting that the theta oscillations detected are related to cognitive functioning. We emphasize the notion that the oscillatory theta activity should be distinguished from the non-rhythmic theta activity identified in relation to other phenomena such as (a) sleepiness and hypoarousal conditions during the waking state and (b) cortical slowing, considered as an EEG alteration in clinical samples.

## Introduction

Several findings revealed that specific electrophysiological (EEG) patterns during sleep are related to the subsequent successful dream recall (DR) upon awakenings (1–7). Bearing in mind the impossibility of a direct access to dreaming, certain studies tried to overcome this limitation analyzing the last segment of sleep and considering DR as the retrieving of an episodic memory trace (2). Following this approach, some investigations reported that the theta (5–7 Hz) (2, 4) and gamma (20–40 Hz) activity (5, 8) during REM sleep predict DR in young subjects.

Although it is well-known that sleep significantly changes across the lifespan (9), the effect of aging on dreaming has been poorly investigated (10). In particular, sleep in older adults is characterized by significant variations in the macro- and micro-structure, such as reductions in total sleep time (TST), sleep efficiency index (SEI), and slow wave activity (SWA), together with increases in wakefulness after sleep onset (WASO), stage 1 and 2 of sleep and a greater number of naps than younger groups (9). Results on REM sleep are mixed: on the one hand, age-related changes in REM sleep have been observed both concerning the duration (9) and some REM sleep features [e.g., reduced eye rapid movements; (11)]. However, REM sleep seems to be affected by significant modifications when subjects have 80 years of age or older (12) or in relation to pathological conditions including degenerative dementias and cognitive decline (13–15).

Taking into account the relation between EEG rhythms and dream experience (10, 16, 17), these sleep changes could affect DR. Some evidences point in this direction, since a general reduction in dream recall frequency (DRF), as evaluated by retrospective questionnaires, has been found in older adults (18–22). However, this finding has been confuted by a longitudinal study (23) and a decrease of DRF has also been found in middle-adulthood (28–38 years) (19, 20). Notwithstanding, it is surprising that, to date, the effect of aging on dreaming has been so poorly investigated with an EEG approach (10). A notable exception is represented by the study by Chellappa et al. (24) reporting that DR during NREM sleep in older compared with young people is related to higher frontal delta activity and centro-parietal sigma activity. Conversely, REM sleep did not show any differences between recall (REC) and non-recall (NREC) conditions (24). These results are not in line with previous findings on young individuals and are challenging to interpret in the existing literature on dreaming.

In order to fill some of the gaps in our knowledge about DR during aging, our study aimed to investigate whether specific EEG oscillations during sleep in the elderly may predict a subsequent dream experience. Although robust evidence showed that dream experience can occur also during NREM sleep [e.g., (5, 6, 25)], here, we focused on DR from REM sleep awakenings, since the EEG background in this stage—mainly characterized by theta and alpha oscillations—could represent a privileged *scenario* to encoding and/or consolidate the mental sleep activity related to cognitive processing.

## Materials and Methods

### Subjects

Twenty healthy young adults (12 M, 8 F; age range 18–29; mean age: 23.4 ± 2.76 SD) and 21 healthy older volunteers (14 M, 7 F; age range 60–79; mean age: 69.2 ± 6.07 SD) participated in the study. Young subjects were recruited among university students. Elderly participants were recruited in clubs for retired people. The exclusion criteria for all subjects were as follows: neurological and/or psychiatric disorders, sleep disturbances or excessive daytime sleepiness, obesity, and history of substance abuse. Sleep quality and sleep habits were assessed by a clinical interview at the recruitment stage.

Informed consent was signed from all elderly and young participants. The research protocol received the approval from the Institutional Ethics Committee of the Department of Psychology of the University of Rome Sapienza (#1128/2016) and adhered to the Declaration of Helsinki.

### Study Design

The young and elderly subjects were requested to respect a regular sleep–wake rhythm during the week before the experimental session and to complete each morning brief sleep log to control their compliance.

Each participant came to the laboratory at 8.00 p.m. and electrodes were fixed on their head and face in about 2 h. Polysomnography (PSG) was recorded in a sound-proof, temperature-controlled room. The recording session started according to the participant's usual sleep schedule. The subjects' sleep was undisturbed. An expert sleep researcher monitored the PSG recording online and chose the moment to wake up the subject when no stage shift over the last 5 min of sleep was observed. The stability of the REM sleep interval was then validated by an expert sleep researcher. The mean time of awakening in elderly is 05:23 (±41 min SD), while in young subjects, it is 7:21 (±38 min SD).

Subjects were awakened in the morning by calling out their first name and coming into the sleep room. Participants were asked to fill out a sleep and dream diary (4, 25, 26), just after the morning awakening. The diary collected information on subjective estimates of sleep quality [i.e., sleep onset latency (SOL), TST, number of awakenings] and of DR (i.e., the presence/absence of DR and the rate of dreams during the preceding night). Preliminarily, each participant was trained to consider all kinds of mental activity occurring during sleep like a dream experience.

White dreams (i.e., the feeling of having a dream but without recalled any item/content) were not taken into account separately from non-recall (NREC) condition. Hence, in our protocol, the NREC was defined as the total absence of dream reports.

### Polysomnographic Recordings

PSG signals were acquired by a Micromed system plus digital polygraph. EEG signals were recorded with a sampling frequency of 256 Hz and bandpass filtered at 0.5–30 Hz. There were 19 unipolar EEG scalp derivations (F1, F2, F3, F4, F7, F8, Fz, C3, C4, Cz, P3, P4, Pz, O1, O2, T3, T4, T5, and T6) and A1 and A2 signals from mastoids, according to the international 10–20 system, by using Ag/AgCl electrodes. The ground electrode was placed at Fpz (fronto-polar location). Electrooculogram (EOG) electrodes were placed about 1 cm from the medial and lateral canthi of the dominant eye. Electrode impedance was <5 kΩ.

## Data Analysis

### Sleep Measures

Sleep stages were visually scored in 20-s epochs, according to the Rechtschaffen and Kales criteria (27). Slow-wave sleep (SWS) scoring strictly adhered to the >75-μV amplitude criterion. The following sleep parameters were examined as dependent variables: (a) stage 1 latency; (b) stage 2 latency; (c) REM sleep latency; (d) stage 1, 2; SWS and REM sleep duration (%); (e) WASO in minutes; (f) number of arousals; (g) number of awakenings; (h) TST, i.e., the sum of time spent in stage 1, stage 2, SWS and REM; (i) total bed time (TBT); (j) SEI (TST/TBT %). The scorer considered an awakening when the EMG or EEG activation lasted more than 10 s, while an arousal was identified in correspondence of an EMG activation, which affected the EEG signals for intervals shorter than 10 s.

The PSG measures were compared by two-way between-subjects design analyses of variance (ANOVA) Recall (REC vs. NREC) × Age (Young vs. Older). The false discovery rate (FDR) was computed to correct for multiple comparisons (28).

### Quantitative Analysis of Sleep EEG: Detection of Oscillatory Activity

The PSG signals of the 5 min of REM sleep before the awakening were analog-to-digital converted online with a sampling rate of 256 Hz, and the EEG signals were referenced offline to the mean of the two mastoid channels (A1 and A2). EEG signals were also visually examined for 8-s epochs to remove artifacts. In order to prevent artifacts on the EEG signal due to the rapid eye movements, only tonic REM sleep intervals were further analyzed. The percentage of tonic epochs considered for further analyses within the last 5 min of REM sleep was 42.48% in young REC, 55.55% in young NREC, 42.05% in older REC, and 42.57% in older NREC. The number of epochs analyzed per participant did not differ by group (*p* > 0.15).

Since the well-known fast Fourier transform (FTT) algorithm can fail in detecting EEG oscillatory activity (29), here, the quantitative analysis of the last EEG interval (5 min) of REM sleep was performed through the Better OSCillation (BOSC) detection algorithm (30).

It should be considered that FFT is firstly designed for regular and stationary signals (29, 31). Nevertheless, the EEG signals are rarely stationary (32) and—to some extent—the EEG correlates of DR have been previously associated to an oscillatory (non-stationary) activity (2, 4).

The BOSC method—proposed by Caplan et al. (30)—has been employed to identify delta oscillations recorded by intracranial-EEG (iEEG) within the hippocampus (33), as well as to detect theta oscillations in the neocortex during sleep and the waking state from EEG scalp recordings (30, 34–38). Furthermore, this algorithm has been applied in elderly subjects during wakefulness (39).

It has been suggested that the BOSC algorithm is a compelling method to detect oscillatory episodes, allowing the minimization of some bias across EEG frequencies, brain areas, various stimuli/tasks, as well as different state of consciousness and species (32). It is a powerful algorithm able to discriminate the time intervals of the recordings in which the rhythmic activity results in significant deviations of the spectral power at specific frequencies from the non-rhythmic “background” EEG signal (2, 32).

For a detailed description of the BOSC procedure, please see the original articles (30, 39).

Briefly, the rhythmic episode must be longer than three cycles [duration threshold (DT)] and exceed a power threshold (PT). The PT was defined as follows: (a) a Morlet Wavelet was computed on the EEG signal (6-cycle window) for each frequency bin (1–30 Hz); (b) the background was estimated as colored noise by a linear regression in log–log units on the observed spectrum at each scalp location (29, 30, 35, 36). We defined P_episode_ (f) as the percentage of time in which significant oscillatory activity was detected during the last 5-min REM sleep interval (29, 30, 35, 36).

The analysis was computed on the EEG signals recorded from each scalp location, separately for each frequency of interest (50 logarithmically spaced frequencies) within the 1–30 Hz range. EEG oscillations were averaged across the whole derivations, to detect the frequency peaks of the oscillatory activity associated with DR, separately for young and older subjects.

Two-way between-measures ANOVAs, Recall (REC vs. NREC) × Age (Young vs. Older), were performed for each electrode, separately for each frequency peak. The FDR correction (28) was applied to adjust the α-value for multiple comparisons.

## Results

Twelve out of 20 young subjects were successful in DR (REC; mean number of recalled dreams = 1.6; SD = 0.7) and 8 were not successful (NREC). Ten out of 21 elderly subjects were REC (mean number of recalled dreams = 1.6; SD = 0.8) and 11 were NREC. Statistical comparisons by unpaired *t*-test showed no differences between the number of recalled dreams between young and older adults.

### Sleep Measures

Table 1 reports the results of the two-way Recall × Age ANOVAs performed on the PSG measures. Not surprisingly, macrostructural variables of sleep show a significant main effect of Age (adjusted critic *p* = 0.002), pointing to a pattern of significant differences between the young and elderly group, representing the typical changes on sleep architecture during aging: increased WASO, a steep decrease in the amount of SWS with an increase of Stage 2, and a decrease of REM sleep. Moreover, TST and TBT are significantly shorter in the elderly than in young subjects. Also, there is an increased number of arousals and awakenings in younger subjects. Neither a significant main effect of the Recall factor nor significant Recall × Age interactions have been observed. This implies that there were no remarkable changes in the sleep architecture of the participants grouped as a function of DR (presence/absence).

**Table 1:** Means and standard errors (SE) of the polysomnographic variables during REM sleep in young and older subjects with REC and NREC.

**Variables**	**Recall**			**Age**			**Recall** **×** **Age**				
							**REC**		**NREC**		
	**REC** **(S.E.)**	**NREC** **(S.E.)**	***F*_**1, 40**_** **(*p*)**	**Young** **(S.E.)**	**Older** **(S.E.)**	***F*_**1, 40**_** **(*p*)**	**Young** **(S.E.)**	**Older** **(S.E.)**	**Young** **(S.E.)**	**Older** **(S.E.)**	***F*_**1, 40**_** **(*p*)**
Stage 1 latency (min)	8.64 (1.51)	15.03 (3.61)	2.11 (0.15)	7.97 (1.69)	15.06 (3.30)	3.17 (0.08)	7.15 (2.08)	10.43 (2.16)	9.21 (2.96)	19.27 (5.83)	0.82 (0.37)
Stage 2 latency (min)	11.39 (2.03)	13.95 (2.42)	0.55 (0.46)	12.30 (2.36)	12.84 (2.15)	0.02 (0.90)	12.30 (3.54)	10.30 (1.59)	12.29 (2.88)	15.15 (3.81)	0.55 (0.46)
REM latency (min)	86.82 (7.25)	77.56 (4.77)	1.04 (0.31)	85.33 (6.70)	79.85 (6.17)	0.13 (0.72)	94.30 (10.25)	77.83 (9.98)	71.88 (3.74)	81.70 (7.96)	2.10 (0.16)
Stage 1 (%)	5.66 (0.62)	5.64 (0.70)	0.01 (0.92)	6.31 (0.58)	5.03 (0.70)	1.69 (0.20)	6.64 (0.86)	4.49 (0.79)	5.81 (0.71)	5.59 (1.15)	0.98 (0.33)
Stage 2 (%)	67.62 (2.14)	68.05 (2.00)	0.81 (0.37)	59.80 (1.09)	75.45 (1.19)	**91.13 (<0.001)**	59.88 (1.39)	76.90 (1.79)	59.68 (1.87)	74.14 (1.59)	0.60 (0.44)
SWS (%)	6.71 (1.45)	3.91 (1.16)	1.43 (0.24)	9.33 (1.37)	1.68 (0.74)	**22.90 (<0.001)**	9.86 (1.99)	2.94 (1.46)	8.54 (1.77)	0.54 (0.27)	0.12 (0.73)
REM (%)	19.23 (1.56)	22.32 (1.30)	5.83 (0.02)	24.57 (1.09)	16.94 (1.36)	**22.90 (<0.001)**	23.64 (1.14)	13.93 (2.19)	25.95 (2.13)	19.68 (1.26)	1.06 (0.31)
WASO (min)	54.02 (10.05)	56.50 (46.67)	0.09 (0.77)	31.19 (8.29)	78.01 (9.34)	**13.75 (<0.001)**	36.77 (13.55)	74.73 (12.71)	22.82 (4.11)	81.00 (14.33)	0.61 (0.44)
Arousals (#)	24.05 (2.11)	31.05 (5.15)	3.82 (0.06)	34.25 (4.43)	20.67 (2.56)	**10.66 (0.002)**	26.42 (2.83)	21.20 (3.09)	46.00 (9.04)	20.18 (4.13)	4.13 (0.05)
Awakenings (#)	24.14 (1.96)	24.89 (2.31)	0.59 (0.45)	28.75 (2.15)	20.43 (1.71)	**11.28 (0.002)**	25.75 (2.91)	22.20 (2.54)	33.25 (7.30)	18.82 (2.30)	4.70 (0.04)
TST (min)	382.58 (19.12)	351.35 (20.27)	0.69 (0.41)	445.82 (7.11)	294.10 (13.06)	**93.44 (<0.001)**	453.98 (9.79)	296.90 (15.51)	433.59 (9.03)	291.55 (21.29)	0.24 (0.63)
TBT (min)	446.00 (17.45)	420.37 (12.84)	0.78 (0.38)	486.93 (11.29)	383.83 (10.84)	**40.21 (<0.001)**	500.76 (16.70)	380.30 (16.57)	466.21 (10.25)	387.03 (14.91)	1.72 (0.20)
SEI % (TST/TBT)	85.42 (2.45)	82.57 (3.12)	0.07 (0.80)	92.10 (1.62)	76.48 (2.61)	**24.11 (<0.001)**	91.44 (2.62)	78.20 (3.18)	93.09 (1.12)	74.92 (4.15)	0.59 (0.45)

*The results of the two-way ANOVAs Recall × Age are also reported. To correct for multiple comparisons, the FDR was applied (significant effects in bold; adjusted critic p = 0.002). SWS, slow-wave sleep; REM, rapid eye movement; WASO, wake after sleep onset; TST, total sleep time; TBT, total bed time; SEI, sleep efficiency index. An awakening was scored whenever an EEG/EMG activation occurred lasting more than 10 s. Arousals have been scored whenever an EMG activation affected the EEG recording for periods shorter than 10 s*.

### Detection and Topography of Oscillatory Activity

**Figure 1:**
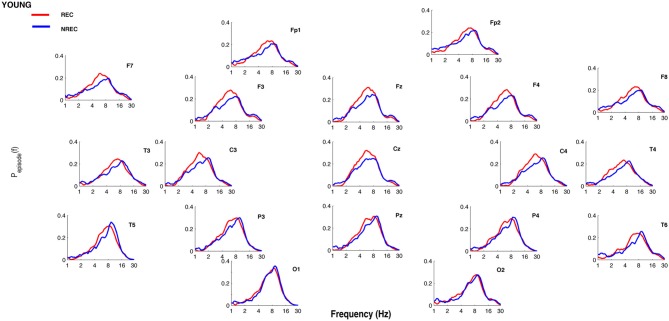
Oscillatory activity in young subjects during the last 5 min of REM sleep. Proportion of time (P_episode_ [f]) occupied by oscillations at each frequency during the last 5 min of REM sleep preceding the awakenings with REC (red line) or NREC (blue line) in young subjects. The mean proportion of time of the EEG activity is plotted at the corresponding scalp location. Units of frequency are expressed in hertz and are plotted in 50 logarithmically spaced frequency values in the 1–30 Hz frequency range.

**Figure 2:**
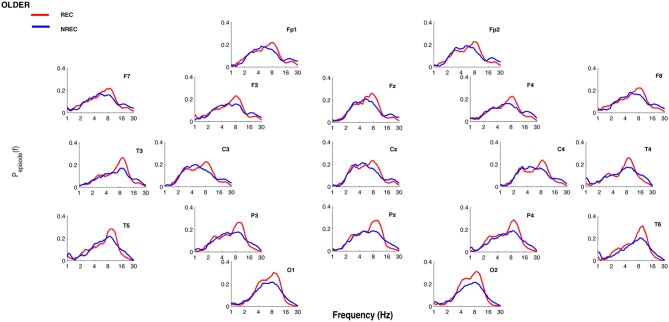
Oscillatory activity in older subjects during the last 5 min of REM sleep. Proportion of time (P_episode_ [f]) occupied by oscillations at each frequency during the last 5 min of REM sleep preceding the awakenings with REC (red line) or NREC (blue line) in older subjects. The mean proportion of time of the EEG activity is plotted at the corresponding scalp location. Units of frequency are expressed in hertz and are plotted in 50 logarithmically spaced frequency values in the 1–30 Hz frequency range.

Figures 1, 2 show the proportion of time (P_episode_ [f]) occupied by oscillations at each frequency during the last 5 min of REM sleep preceding the awakenings with REC or NREC, respectively, in young and elderly subjects. The mean proportion of time of the EEG activity is plotted at the corresponding scalp location, showing a prevalence of theta and alpha oscillations in REC compared to NREC subjects.

**Figure 3:**
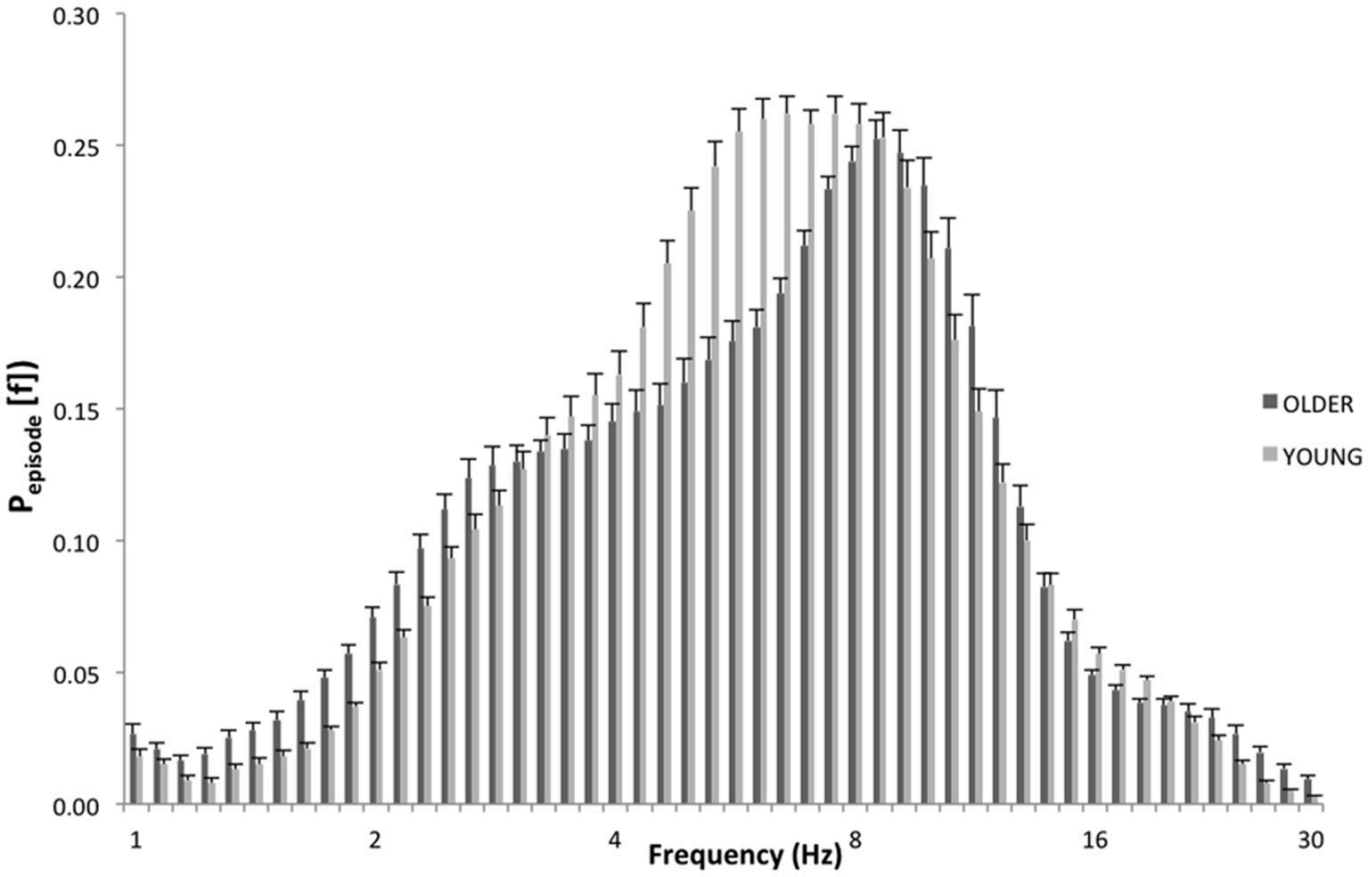
EEG oscillations averaged across the whole derivations during the last 5 min of REM sleep. The figure plots the mean proportion of time (P_episode_ [f]) of the EEG in which oscillations were detected at each frequency for young (light gray) and elderly (dark gray) group with dream experience (REC). The detection of oscillations has been made by the better oscillation detection method on the 19 EEG electrodes. Units of frequency are expressed in hertz and are plotted in 50 logarithmically spaced frequency values in the 1–30 Hz frequency range.

Specifically, as depicted by Figure 3, which details EEG oscillations averaged across all the derivations in the REC condition for the two groups, the EEG recordings associated with dream experience (REC) in young subjects were dominated by theta oscillations peaking at 6.5 and 7.4 Hz. In older subjects, the EEG associated with REC was characterized by a prevalent alpha oscillatory activity, peaking at 8.6 Hz. Since a previous study showed the association between the theta peak at 6.5 Hz (2) and dream experience, we focused on this peak of frequency for the theta range and on the peak at 8.6 Hz for the alpha range.

**Figure 4:**
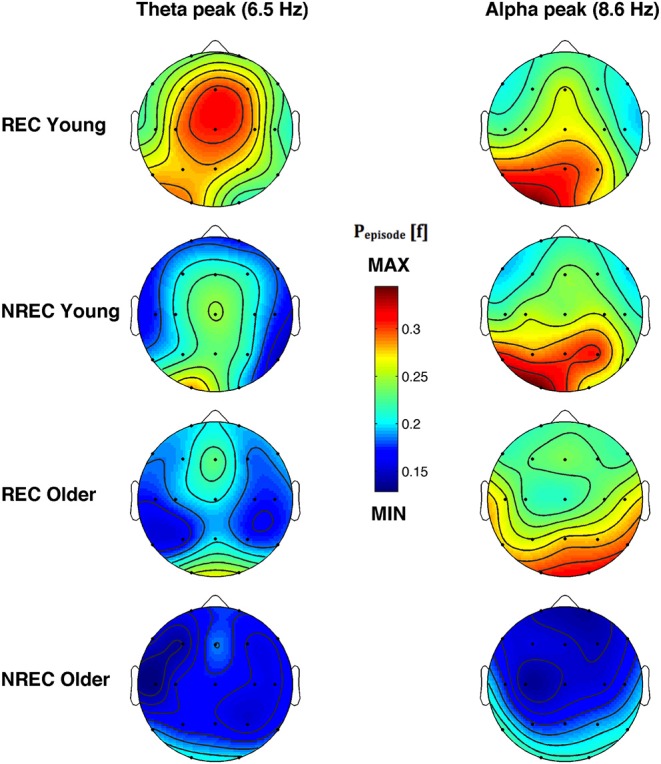
Topographic distribution of the frequency peak of oscillatory activity within theta and alpha range. From the left, the first and the second column show the topographic distribution of the mean proportion of time in which oscillations were detected (P_episode_ [f]) in correspondence of the selected frequency of interest within the theta (6.5 Hz) and alpha (8.6 Hz) range during the last 5 min of REM sleep preceding the awakenings with REC and NREC young subjects (first two rows) and REC and NREC elderly subjects (last two rows). The maps are based on 19 derivations (electrode positions indicated by black dots) of the international 10–20 system with averaged mastoid reference. Values are color-coded and plotted at the corresponding position on the planar projection of the hemispheric scalp model. Values between electrodes were interpolated (biharmonic spline interpolation).

The topographic distribution of the EEG oscillations at the frequency peaks within theta (6.5 Hz) and alpha (8.6 Hz) range is depicted in Figure 4. Theta oscillations showed a stable distribution in all groups, with a peak over centro-frontal and occipital areas. Alpha oscillations also showed a stable distribution in all groups, with a maximum over the parieto-occipital regions. More generally, older subjects with NREC showed the lowest amount of oscillatory activity over the scalp.

**Figure 5:**
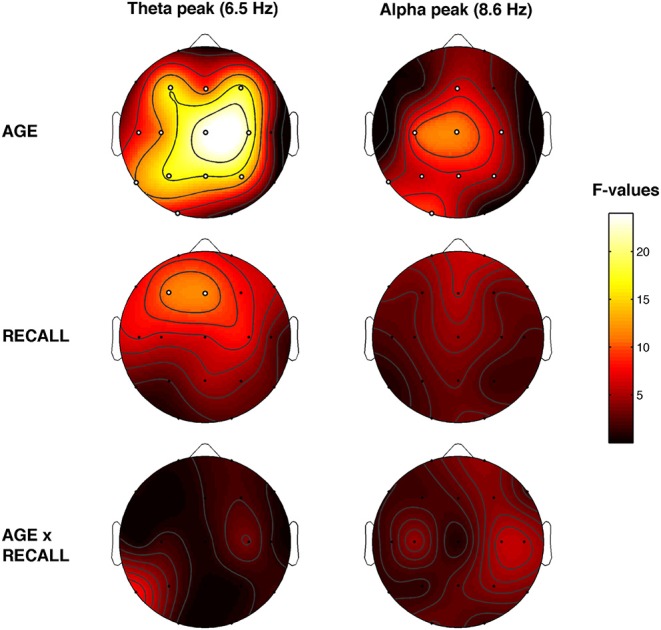
Topographical statistical P_episode_ differences assessed by the two-way ANOVAs *Recall* × *Age*. Statistical maps reporting the results of two-way ANOVAs, Recall (REC vs. NREC) × Age (Young vs. Older) for each selected frequency peak at 6.5 and 8.6 Hz. The main effects are reported in the first two rows, and the interactions are depicted in the third row. Values are expressed in *F*-values. Significant effect of the Age (critic *p* = 0.0272) and Recall factor (critic *p* = 0.002). White dots indicate significant effects after the FDR corrections. The maps are based on 19 derivations of the international 10–20 system with averaged mastoid reference. Values are color-coded and plotted at the corresponding position on the planar projection of the hemispheric scalp model. Values between electrodes were interpolated (biharmonic spline interpolation).

Figure 5 showed the topographical maps of the *F* coefficients from the two-way ANOVAs “Recall × Age” performed for each peak of frequency. The main effect of Age was observed. The α-value after the FDR procedure was adjusted to a critic *p* = 0.0272 for the Age factor. Significant differences were found for 12 out of 19 scalp derivations in the theta range (C3, *F* = 11.53 *p* = 0.0016; C4, *F* = 20.62 *p* = 0.0001; Cz, *F* = 23.97 *p* = 0.0001; F3, *F* = 15.99 *p* = 0.0003; F4, *F* = 17.50 *p* = 0.0002; Fz, *F* = 9.47 *p* = 0.0039; O1, *F* = 6.09 *p* = 0.018; P3, *F* = 16.84 *p* = 0.0002; P4, *F* = 14.43 *p* = 0.0005; Pz, *F* = 16.34 *p* = 0.0003; T3, *F* = 8.07 *p* = 0.0073; T5, *F* = 11.63 *p* = 0.0016) and for nine derivations in the alpha range (C3, *F* = 9.41 *p* = 0.004; C4, *F* = 5.77 *p* = 0.0214; Cz, *F* = 11.48 *p* = 0.0017; Fz, *F* = 6.53 *p* = 0.0148; O1, *F* = 9.58 *p* = 0.019; P3, *F* = 6.01 *p* = 0.0123; P4, *F* = 5.33 *p* = 0.0266; Pz, *F* = 6.92 *p* = 0.0123; T5, *F* = 5.29 *p* = 0.0272). Theta and alpha oscillations are greater in young compared to older adults.

Also, the main effect of Recall was observed. The α-value after the FDR procedure was adjusted to a critic *p* = 0.002 for the Recall factor. Significant differences in the theta range were found over the frontal area (F3, *F* = 11.12 *p* = 0.002 and Fz, *F* = 11.67 *p* = 0.0016). The dream experience was related to a greater amount of theta oscillations over the frontal area compared to NREC. No significant effect was found on alpha range (all *p* ≥ 0.018). No significant interaction has been observed.

The results have been substantially confirmed by the statistical analysis (Recall Age ANOVAs) on the theta (4.6–7.5 Hz) and alpha (8–11.3) bands, obtained averaging across frequency bins (see Figure S1).

## Discussion

Along with the expected differences between older and young subjects (9), we showed for the first time the EEG correlates of DR in elderly people after a regular night of sleep.

Specifically, the detection of oscillatory activity revealed that older as well as young individuals recall their dream experience when the last segment of REM sleep is characterized by frontal theta oscillations. Not surprisingly, this result replicated the previous evidence in healthy young subjects, as shown in within-subjects (4) and between-subjects (2) design.

Our results are completely original for older individuals, demonstrating that theta oscillations are crucial for the retrieval of dreaming also in this population. It should be noted that theta oscillations are of particular interest for cognitive functions during the waking state, such as for spatial memory, episodic memory, and motor tasks and are related to synaptic plasticity (40). The theta increases for retrieved items/contents have been demonstrated for different sets of stimuli and tasks, and it has been proposed that the theta oscillations reflect several processes involved in the declarative memory both in young and elderly people (41, 42). Theta oscillations are also associated with the hippocampal activity (43), promoting memory formation and encoding, as confirmed by iEEG studies (44, 45). Moreover, the theta rhythm modulates the interaction between other areas contributing in the recall processes, such as the medial temporal lobe and the medial prefrontal cortex (46).

In this vein, we point out that DR can be considered as retrieving of a memory trace mediated by theta oscillations, since the same EEG pattern associated with good memory performance during wakefulness is necessary during REM sleep to retrieve the dream experience. In other words, our results are in line with the “Continuity Hypothesis” between waking and sleep mental functioning from a neurobiological viewpoint (2).

Moreover, consistently with previous data from the BOSC analysis on the EEG signals during the resting state, we did not observe a greater presence of theta oscillations in healthy aging during REM sleep (39). Conversely, we found a greater amount of rhythmic theta and alpha activity in the REM sleep of young than older participants. In this context, it is worth underlining that oscillatory and non-oscillatory cortical activity could play different functional roles in cognitive processing. Indeed, non-rhythmic theta activity has been identified in relation to phenomena such as (a) sleepiness and hypoarousal conditions during the waking state (47) and (b) cortical slowing, an EEG signature of neurocognitive decline, such as in Alzheimer's disease (39, 48), while it is the oscillatory component of the cortical activity in the theta band that seems to mediate the memory processes. Accordingly, our focus on cortical oscillations should be considered as a further strong point of our study and, in this perspective, we point out the importance to distinguish rhythmic from non-rhythmic component of cortical activity when it is looking to cognitive processes.

It is worth noting that when the comparison on alpha oscillations was performed considering the older group only, significant difference between REC and NREC was found in a widespread manner over the scalp, with a maximum difference over centro-frontal regions, in the direction of higher alpha oscillations in REC compared to the NREC group (*t* > 2.29, *p* < 0.0338; data not shown). Although no interaction was found, we can speculate that alpha oscillations may also have a role in retrieving the dream experience in older adults. It should be noted that several studies found that the fluctuations in the alpha activity during wakefulness reflect—to some extent—the memory processes [for a review, see (49)]. In this regard, the results appear more heterogeneous for what concerns the direction of topographic distributions during sleep. For instance, lower alpha oscillations during REM (1) and NREM sleep (1, 2) have been related to DR. More directly, Chellappa et al. (50) found that lower frontal alpha along with high occipital alpha activity is related to dream experience during REM sleep in young subjects. Also, during wakefulness, occipito-parietal alpha oscillations have been associated to mental imagery, when subjects were asked to image words with emotional contents (51). In addition, recent studies posited that the frontal alpha asymmetry in waking state is related to emotional dreams during REM sleep (52).

Albeit our results confirm previous findings on young adults for what concerns the theta activity (2, 4, 7, 53), we have to mention some discrepancies with other studies investigating DR from REM sleep awakenings, since they failed to find a relationship between DR and REM theta activity (1, 3, 50). Besides the abovementioned studies by Chellappa et al. (50) and Esposito et al. (1), Takeuchi et al. (3) showed that the absence of dream after REM awakenings is related with increased central alpha and sigma compared to recall condition. The heterogeneity of these results may be ascribed to the different protocols. For instance, the time of awakening differs among studies. Indeed, DR was collected after early and late REM episodes (1), multiple nap across 40 h under constant routine (50) or REM sleep onset periods (3). We cannot rule out that circadian and homeostatic factors could have affected the EEG correlates of DR (54), explaining the differences among findings.

In particular, it should be noted that while the theta activity has been suggested as state-dependent (4), the differences on alpha oscillations among studies (1, 3, 50) could be ascribed—more likely—to trait-like factors (55, 56). In this view, we suggest that the interindividual differences on alpha band should explain the absence of significant results on the total sample (young and older). Moreover, we point out that the fluctuations in the alpha range should be studied by analyzing the individual frequency peak of the subjects recorded.

Actually, the different approach in the signal processing (FFT vs. BOSC detect method) could account for the inconsistency between our results and those reported by Chellappa et al. (24), which found no differences between older subjects with REC and NREC by using the traditional FFT, since it could be ineffective to detect rhythmic oscillations (39). Furthermore, it is worth noting that their protocol consisting of multiple naps during 40 h may have affected those results, introducing some problems related to homeostatic and circadian factors.

Albeit we did not collect the DRF for several days, the DR rate of our older sample did not differ from the DR rate of younger subjects, in contrast with the findings of a general reduction in dreaming in elderly people (18–22). In this respect, we have to consider that the participants were instructed before the goodnight to consider any distinct mental activity occurring during sleep as a dream and this procedure could increase the attention on their dreams. In fact, some authors hypothesized that the decline in DRF among the elderly could be ascribed to a reduction in dream salience; in other words, older people show a diminished interest in dream contents (19, 57). Consistently, a study showed that when elderly people were motivated to remember their mental sleep activity, the DRF increased (58).

## Limitations and Conclusions

To the best of our knowledge, this is the first study investigating the EEG oscillations related to DR during REM sleep in elderly people. Furthermore, our protocol allows collecting REC or NREC after a total sleep night avoiding the interferences by circadian and homeostatic variables. Nevertheless, some limitations should be addressed. Firstly, we collected dream reports with the aim of confirming the presence/absence of DR, without any possibility of analyzing the qualitative features of dreaming that could change during aging (10). Moreover, we did not consider the “white dreams,” and this means that we are unable to disentangle whether subjects did not report any dreams (REC condition) because they could not retrieve the oneiric contents or because they did not dream at all.

Secondly, our study is based on a between-subjects design and—albeit also intraindividual measures (4) revealed difference on theta oscillation—we cannot rule out the interference of trait-like features of the subjects on our results [for a review, see (16)]. This is also true for what concerns our negative finding on oscillations in the alpha band that are affected by a strong inter-individual variability (56).

Besides, we were unable to analyze the gamma oscillations, since the signals have been filtered at 30 Hz. Previous studies highlighted that higher rapid frequencies could be related to dream experience (5, 8); hence, further investigations should apply the algorithm to detect oscillatory activity also on gamma band.

Finally, we did not record an adaptation night, especially because of the difficulty of older adults to change their habits and to sleep outside their house for consecutive days. In this view, future studies should provide PSG home recordings in order to avoid the “first night effect” and confounding variables related to the discomfort of elderly subjects.

To sum up, our results confirmed the pivotal role of theta oscillations in DR, showing that this relation is maintained also in healthy older adults. This evidence is in line with the idea that shared mechanisms are involved in cognitive and memory processing between sleep and wakefulness, as suggested by several authors (2, 16, 17, 59, 60).

Bearing in mind that rhythmic oscillatory activity could represent an index of cognitive encoding (30, 39), the preserved ability to remember dreams with the same mechanisms of young adults allows us to hypothesize that dreaming—to some extent—is an expression of cognitive functioning. In this vein, some evidence point out that the decline of DR rate parallels the cognitive decline (61); hence, further studies should be addressed whether changes in EEG correlates of DR could mirror the cognitive decline in patients affected by neurodegenerative syndromes.

## Data Availability Statement

The datasets generated for this study are available on request to the corresponding author.

## Ethics Statement

The studies involving human participants were reviewed and approved by Institutional Ethics Committee of the Department of Psychology of the University of Rome Sapienza (#1128/2016). The patients/participants provided their written informed consent to participate in this study.

## Author Contributions

SS and LD: substantial contributions to the conception and design of the work, interpretation of data, and drafting the work and revising it critically for important intellectual content. SS, CB, AM, AD'A, and MG: acquisition and analysis of data. SS, AD'A, CB, AM, MG, and LD: final approval of the paper and agreement to be accountable for all aspects of the work in ensuring that questions related to the accuracy or integrity of any part of the work are appropriately investigated and resolved.

### Conflict of Interest

The authors declare that the research was conducted in the absence of any commercial or financial relationships that could be construed as a potential conflict of interest.
