# Supplementary material for: Oscillatory EEG Activity During REM Sleep in Elderly People Predicts Subsequent Dream Recall After Awakenings
Source: Front Neurol. 2019 Sep 20;10:985. doi: 10.3389/fneur.2019.00985 (PMC6763554; doi:10.3389/fneur.2019.00985)
Supplement: Supplementary file 1 [file Data_Sheet_1.PDF]

Supplementary Material

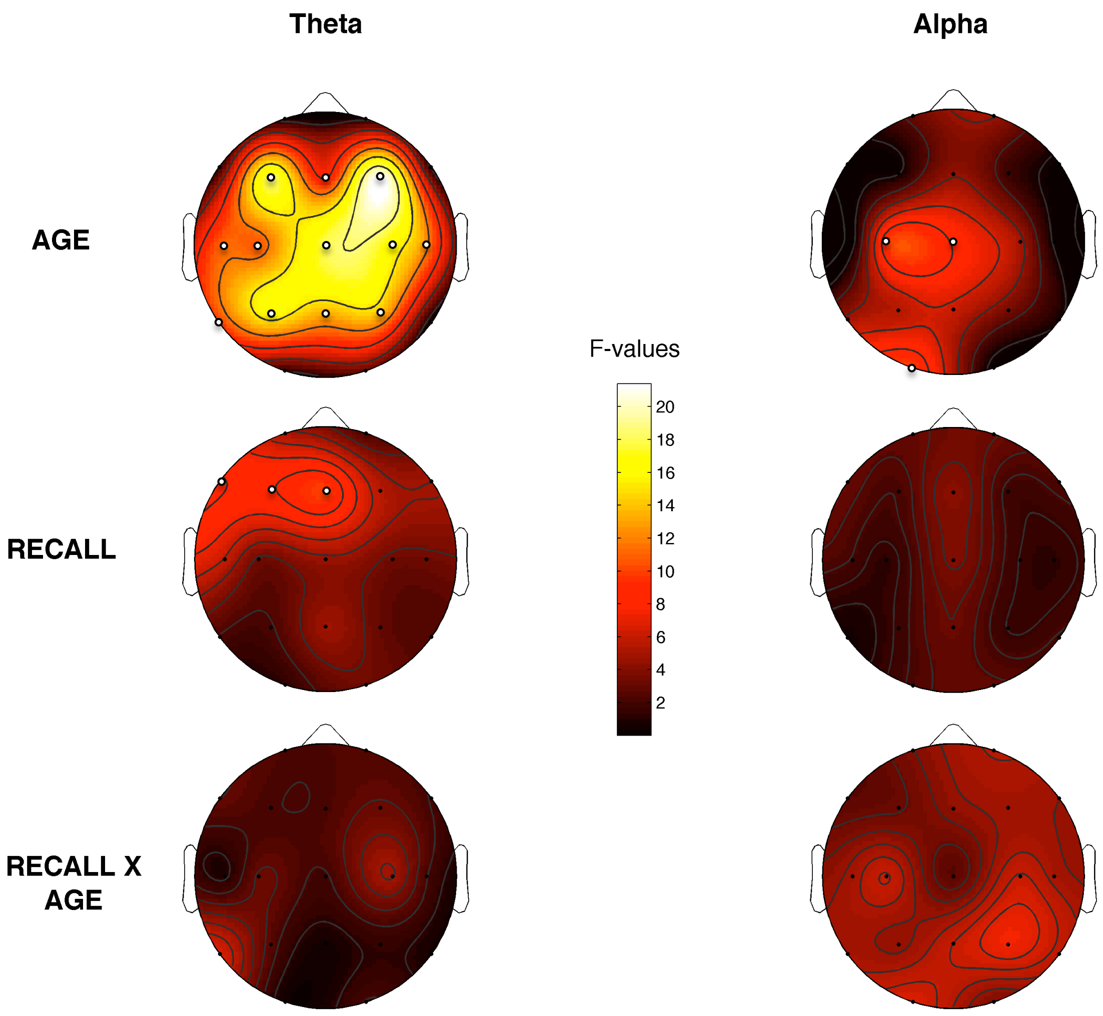

Figure S1. Topographical statistical band differences assessed by the two-way ANOVAs *Recall x Age.*

Statistical maps reporting the results of two-way ANOVAs, Recall (REC vs. NREC) x Age (Young vs. Older) for theta (4.6-7.5 Hz) and alpha (8-11.3) bands. The main effects are reported in the first two rows. and the interactions are depicted in the third row. Values are expressed in F-values. Significant effect of the Age (*critic p=0.0076*) and Recall factor (*critic p=0.006*). White dots indicate significant effects after the FDR corrections. The maps are based on 19 derivations of the international 10-20 system with averaged mastoid reference. Values are color-coded and plotted at the corresponding position on the planar projection of the hemispheric scalp model. Values between electrodes were interpolated (biharmonic spline interpolation)

Factor AGE - THETA

C3 F=10.69 p=0.002

C4 F=16.17 p=0.0003

Cz F=17.86 P=0.0001

F3 F=18.01 p=0.0001

F4 F= 21.38 p<0.0001

Fz F= 7.97 p=0.0076

P3 F= 16.17 p=0.0003

P4 F= 13.88 p=0.0006

Pz F= 13.55 p=0.0007

T3 F= 11.10 p=0.002

T4 F= 12.17 p=0.001

T5 F= 9.88 p=0.003

Factor AGE - ALPHA

C3 F= 9.50 p=0.0039

Cz F= 8.34 p=0.006

O1 F= 9.85 p=0.0033

Factor RECALL - THETA

F3 F=8.50 p= 0.006

F7 F=8.70 p= 0.0055

Fz F=9.81 p= 0.0034
